# A cluster randomized trial of delivery of intermittent preventive treatment of malaria in pregnancy at the community level in Burkina Faso

**DOI:** 10.1186/s12936-020-03356-9

**Published:** 2020-08-05

**Authors:** Julie R. Gutman, Daniel K. Stephens, Justin Tiendrebeogo, Ousmane Badolo, Mathurin Dodo, Danielle Burke, John Williamson, Kristen Vibbert, Susan J. Youll, Yacouba Savadogo, William R. Brieger

**Affiliations:** 1 Malaria Branch, Division of Parasitic Diseases and Malaria, Center for Global Health, US Centers for Disease Control and Prevention, 1600 Clifton Road, Mailstop H24-3, Atlanta, GA 30329 USA; 2 The Johns Hopkins Bloomberg School of Public Health, Johns Hopkins University, Baltimore, MD USA; 3 Jhpiego, Ouagadougou, Burkina Faso; 4 Jhpiego, Baltimore, MD USA; 5 US President’s Malaria Initiative, US Agency for International Development, Washington, D.C. USA; 6 National Malaria Control Programme, Ministry of Health, Ouagadougou, Burkina Faso

**Keywords:** Malaria, Pregnancy, Intermittent preventive treatment, Sulfadoxine–pyrimethamine

## Abstract

**Background:**

Malaria in pregnancy is responsible for 8–14% of low birth weight and 20% of stillbirths in sub-Saharan Africa. To prevent these adverse consequences, the World Health Organization recommends intermittent preventive treatment of pregnant women (IPTp) with sulfadoxine–pyrimethamine be administered at each ANC visit starting as early as possible in the second trimester. Global IPTp coverage in targeted countries remains unacceptably low. Community delivery of IPTp was explored as a means to improve coverage.

**Methods:**

A cluster randomized, controlled trial was conducted in 12 health facilities in a 1:1 ratio to either an intervention group (IPTp delivered by CHWs) or a control group (standard practice, with IPTp delivered at HFs) in three districts of Burkina Faso to assess the effect of IPTp administration by community health workers (CHWs) on the coverage of IPTp and antenatal care (ANC). The districts and facilities were purposively selected taking into account malaria epidemiology, IPTp coverage, and the presence of active CHWs. Pre- and post-intervention surveys were carried out in March 2017 and July–August 2018, respectively. A difference in differences (DiD) analysis was conducted to assess the change in coverage of IPTp and ANC over time, accounting for clustering at the health facility level.

**Results:**

Altogether 374 and 360 women were included in the baseline and endline surveys, respectively. At baseline, women received a median of 2.1 doses; by endline, women received a median of 1.8 doses in the control group and 2.8 doses in the intervention group (p-value < 0.0001). There was a non-statistically significant increase in the proportion of women attending four ANC visits in the intervention compared to control group (DiD = 12.6%, p-value = 0.16). By the endline, administration of IPTp was higher in the intervention than control, with a DiD of 17.6% for IPTp3 (95% confidence interval (CI) − 16.3, 51.5; p-value 0.31) and 20.0% for IPTp4 (95% CI − 7.2, 47.3; p-value = 0.15).

**Conclusions:**

Community delivery of IPTp could potentially lead to a greater number of IPTp doses delivered, with no apparent decrease in ANC coverage.

## Background

Despite the considerable decline in malaria since 2000, malaria in pregnancy (MIP) remains a major public health problem. MIP is associated with maternal anaemia, preterm deliveries, low birth weight, and an increased risk of neonatal death [1]. Reference before punctuation, please change throughout to prevent the adverse effects of MIP, in areas of moderate to high malaria transmission, the World Health Organization (WHO) recommends the use of long-lasting insecticide-treated nets (LLINs), intermittent preventive treatment of malaria in pregnancy (IPTp) with sulfadoxine pyrimethamine (SP), and prompt and effective treatment of pregnant women, targeting 100% coverage among at risk populations [2–4].

Since 2012, the WHO has recommended that pregnant women receive IPTp-SP as early as possible starting at the beginning of the second trimester (13th week of pregnancy), at every antenatal care (ANC) contact (which typically occurs in health facilities) up until delivery, with doses spaced at least 1 month apart, ideally administering a minimum of three doses during pregnancy (IPTp3) [5]. Despite this recommendation, which was meant to increase IPTp uptake to 3 or more, coverage remains low. In 2017, among 33 African countries, an estimated 22% of pregnant women received the recommended three doses, and only 42% received two doses [6]. Novel strategies are needed to improve uptake.

Several studies have suggested that community health workers (CHWs) might be able to effectively deliver IPTp while at the same time promoting ANC attendance at health facilities [7–11]. Two studies in Nigeria and Uganda utilized CHWs who were integrated with local health services and trained to deliver IPTp and refer women to ANC. These studies improved uptake of at least two doses of IPTp (by 35.3 percentage-points in Nigeria and 37.3 percentage-points in Uganda); in Nigeria there was no effect on ANC attendance, while in Uganda, mean ANC attendance was 3.3 visits in the intervention arm and only 2.6 in the control arm (p < 0.001) [7, 8]. Two studies in Uganda and Malawi trained a variety of community-based agents to deliver IPTp, without explicitly seeking to increase ANC attendance, and while IPTp uptake improved (27.6 and 29.3 percentage point difference in Uganda and Malawi, respectively), ANC attendance did not (19.3 and 17.9 percentage point lower ANC attendance in the community delivery arm in Uganda and Malawi, respectively) [9, 10]. A prior study in Burkina Faso used female community volunteers to encourage both ANC attendance and IPTp uptake through ANC, resulting in increased ANC attendance (19.5 percentage points higher in the community promotion arm) and IPTp uptake (22.7 percentage points higher in the community promotion arm) [11].

In its 2016–2020 National Malaria Strategic Plan, Burkina Faso set a goal of 100% of pregnant women receiving three doses of IPTp [4]. However, coverage of IPTp3 remains below national and international targets at 57.7% [12], despite high rates of ANC attendance (95% of women attend at least once and 33.7% attend four times) [13]. Thus, the Ministry of Health (MOH) is exploring alternative approaches to providing IPTp, including community level distribution of IPTp (cIPTp).

## Methods

A cluster randomized, controlled trial assessing the effect of IPTp administration by CHWs (compared to the routine practice of delivering IPTp at health facilities) on the coverage of IPTp and ANC was conducted in three districts of Burkina Faso over 15 months, from May 2017 to August 2018, with baseline and post-intervention surveys carried out in March 2017 and July–August 2018, respectively.

### Study area

**Fig. 1:**
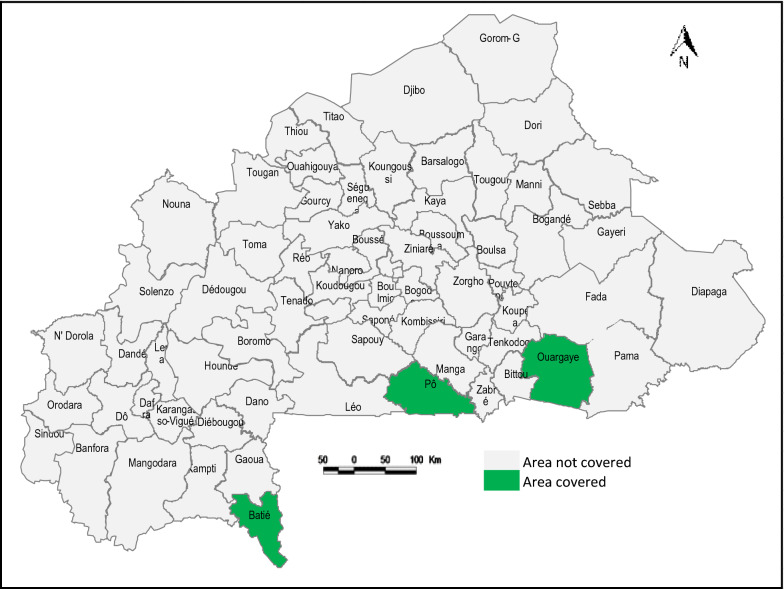
Map of Burkina Faso showing the three districts involved in the study

With an estimated population of 20 million, Burkina Faso is a high malaria endemic Sahelian country in the heart of West Africa accounting for 4% of the world’s annual malaria cases [14]. Women comprise 51.8% of the overall population, and it is expected that 5.5% of women will be pregnant at any given time [15]. The study was implemented in three of the 13 regions (*Sud*-*Ouest* [South-West], *Centre*-*Sud* [Central-South], and *Centre*-*Est* [Central-East]) with highest malaria transmission. One district was purposively selected from each of the three regions, taking into account malaria epidemiology, IPTp coverage, and the presence of active CHWs (Fig. 1). Routine HMIS data indicate that before the trial, IPTp3 coverage in the selected districts, Batié, Pô, and Ouargaye, was 34.6%, 52.8%, and 47.2%, respectively, for an overall average of 44.9%.

Non-contiguous health facilities (HF) in each district were matched by estimated general population and estimated number of pregnant women and IPTp2 coverage (based on routine health facility data). In each district, two pairs of HFs were purposively selected, and one HF in each pair was randomly assigned to the intervention, for a total of 12 HFs (six intervention, six control) (Table 1).

**Table 1:** Characteristics of included facilities

District	Study arm	Health facility	Total population	Number of villages	Number of community health workers	Number of expected births annually
Batié	Intervention	Dankana	6991	21	42	379
	Control	Midebdo	7648	30	60	417
	Intervention	Boussoukoula	5484	16	32	297
	Control	Tamipar	6078	16	32	332
Pô	Intervention	Guiaro	9001	8	8	490
	Control	Kaya	8408	6	6	458
	Intervention	Kampala	7760	8	8	423
	Control	Guelwongo	8757	3	3	477
Ouargaye	Intervention	Nabangou	5770	4	12	334
	Control	Salembore	6467	2	12	374
	Intervention	Konglore	3490	2	6	202
	Control	Tensobtenga	4012	1	8	232

The study intervention included all pregnant women living in the targeted areas, while the baseline and endline surveys included women aged 18–49 years in selected households who had given birth in the 9 months prior to each survey. The catchment area of these HFs included 113 villages, all of which were included in the cross-sectional survey, with sample size per village selected proportional to village size. At the village level, the Expanded Programme on Immunization (EPI) sampling method was used to select households where women would be interviewed [16]. At the center of the village, a direction was chosen by spinning a bottle on the ground. Households along the indicated path were visited and the questionnaire was administered to consenting, eligible women until the desired sample size was achieved.

### Sample size

A sample size of 360 women (30 women per health facility catchment area) who had delivered in the last 9 months was required for each survey to achieve 80% power to detect a difference between the group proportions of approximately 19.6%, from a baseline proportion of 44.7% (average proportion in the three districts) to an endline proportion of 64.3% in the intervention arm, as estimated using PASS V14 (NCSS, LLC, Kaysville, UT) to assess sample size for cluster randomized trial, at a significance level of 0.05 and assuming an intra-cluster correlation (ICC) of 0.03.

### Implementation process

The CHW registers were designed to provide a reminder that IPTp should be given monthly, and not more frequently, and to help the CHW reconcile who needed follow-up. Existing male CHWs were paired with female volunteers (“*animatrices*”), to ensure that home visits were only conducted by women, as there was concern that it would not be culturally acceptable for male CHWs to enter the homes and speak to pregnant women. If the female volunteer was not literate, the male CHW in that village assisted her in completing the forms, and at times accompanied her on the home visit. Formal CHWs received approximately $35USD monthly from the government as compensation for their duties; female volunteers recruited by the study received the same amount in monthly compensation from the study.

The MOH ensured the availability of SP tablets/treatment for IPTp in both control and intervention HFs throughout the study, including for distribution by trained CHWs. Each pregnant woman seen at ANC in the intervention facility received information on the community IPTp study and had the choice to participate or not. Pregnant women who wanted to receive SP had their identities recorded on a summary sheet and in CHW registers to facilitate their follow-up for the intervention. Women were asked to follow-up with the CHW monthly; those who did not present for the scheduled visit with the CHW were followed at home. The first dose of IPTp-SP was always given as directly observed therapy (DOT) at the HF to ensure that a health provider confirmed the woman’s gestational age and initiated IPTp after the end of the first trimester. CHWs also provided IPTp-SP as directly observed therapy, as part of their monthly household visits starting with the second dose, provided that SP had not been administered in the preceding 4 weeks. This was then recorded on the woman’s ANC card, which had space for recording up to five doses. CHWs also encouraged women to attend ANC, collected data on any adverse events, and referred women to the facility in the case of adverse events. CHW data were collected monthly. CHWs were supervised monthly by HF workers; monthly meetings were facilitated by the study.

### Training

Activities were implemented using the cascade training approach. The first training session included central-level stakeholders (National Malaria Control Programme, Directorate of Family Health, Directorate for Health Promotion and Education, Directorate of Sectorial Statistics) and the field coordinator from each district. The main objective of this training was to orient stakeholders on the organization and use of the various intervention tools. The stakeholder then transferred the information to the HF health workers, who in turn trained the CHWs (33 existing male and 25 existing female CHWs plus an additional 33 female volunteers).

For each survey, ten people were trained over 2 days in each district, and eight were chosen as field data collectors. The trainings focused on mastery of data collection tools, ethical rules, and ensuring data quality. CHWs and HF workers were trained on potential adverse events related to SP administration and instructed to report any adverse events to the study coordinator.

### Data analysis

Routine service data (ANC visits, IPTp doses delivered by CHWs and at the HF) were summarized monthly to monitor the intervention. The primary outcome was the change in IPTp3 coverage over time assessed using a difference in difference analysis comparing the baseline and endline cross sectional survey data. In addition, a descriptive analysis of the socio-demographic characteristics of women included in the baseline and endline surveys, stratified by intervention and control areas, was performed. The REG procedure in SAS was used to assess for differences in medians. Difference in differences analyses were conducted on the survey data to assess the change over time in additional secondary outcomes, including any ANC visits (ANC1), four or more ANC visits (ANC4), IPTp1, 2, 4, and 5 coverage. Both primary and secondary outcomes were based on what was recorded on the ANC card, up to a maximum of five IPTp doses. The analyses used logistic regression with generalized estimating equations with the GENMOD procedure in SAS Version 9.4 (SAS Institute Inc., Cary, NC) for binary outcomes (ANC1, ANC4, IPTp3, IPTp4) with difference in differences calculated using a binary model with an identity link function, and Poisson regression for continuous outcomes (number of IPTp doses, number of ANC visits, timing of initiation of ANC). This was done by incorporating an intervention group variable, a variable indicating pre- or post-intervention, and an intervention-time interaction term. The ICC was calculated using the same model with the “corrw” option in the repeated statement. In order to obtain 95% confidence intervals (95% CI) for the difference in differences, a linear model was run using the GENMOD procedure, including all the same terms as in the logistic model. All analyses accounted for clustering at the HF level. Maternal age and gravidity were included a priori in the model; district and maternal education were explored as covariates but dropped due to lack of significance. A p-value of less than 0.05 was considered statistically significant.

### Ethics

The protocol was reviewed and approved by the John’s Hopkins School of Public Health (JHSPH) and Burkina Faso Institutional Review Boards (IRB); the Centers for Disease Control and Prevention Human Subjects Office determined that CDC staff were not engaged in human subjects research. Verbal informed consent was obtained from each woman in the local language prior to data collection.

## Results

### Routine data

**Fig. 2:**
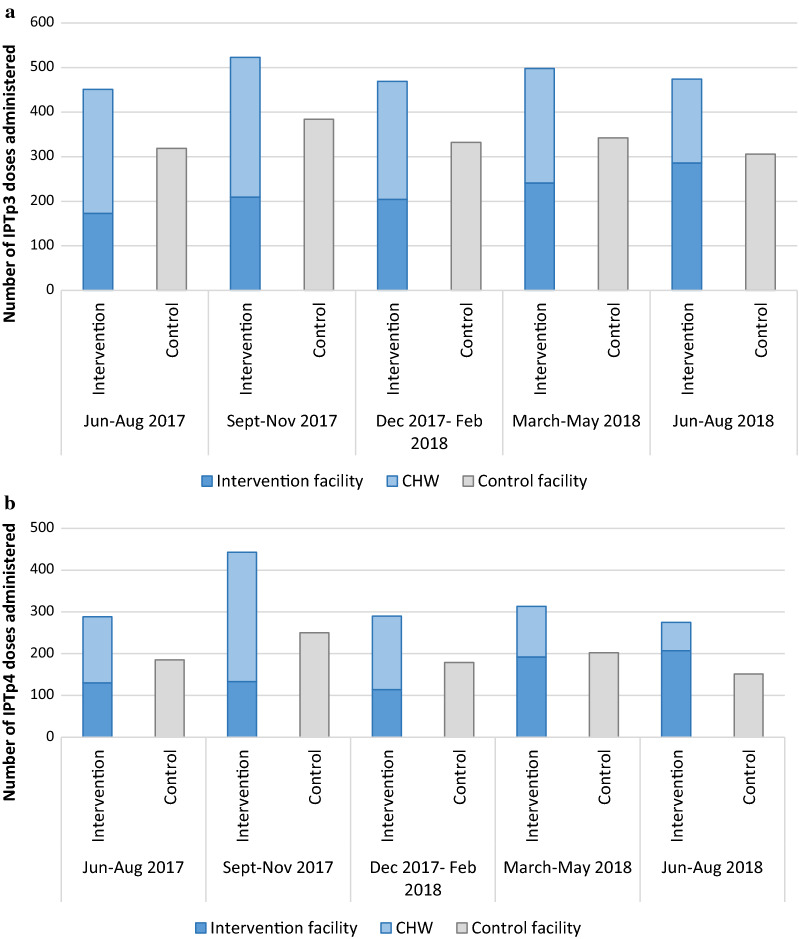
Number of **a** IPTp3 and **b** IPTp4 doses administered by district and quarter from June 2017 to August 2018Coverage rates for IPTp3 (**a**) and IPTp4 (**b**) based on routine health facility and CHW data

**Fig. 3:**
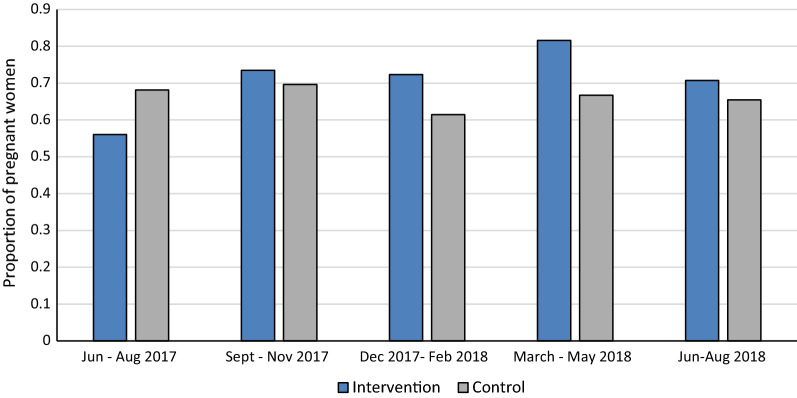
Proportion of women retained in ANC4 (ANC4/ANC1) by study arm and quarter

Overall, 9834 doses were delivered to 2266 women living in the intervention HF catchment areas, with 50% of these given by CHWs, while 6683 doses were delivered to 2030 women living in the control HF catchment areas. There was improvement in the number of doses of IPTp delivered (Fig. 2), as well as in retention in care, measured by the number of women who completed four or more ANC visits out of the number of women who attended the first ANC visit (Fig. 3). During the period of the study, no adverse events related to SP administration were reported.

### Cross sectional survey results

A total of 374 women were interviewed in the baseline survey and 360 in the endline survey. Socio-demographic characteristics of women in the control and intervention groups were similar both within and between surveys (Table 2). On average, a quarter of surveyed women had had one pregnancy, one-third had two to three pregnancies, and the rest had four or more.

**Table 2:** Sociodemographic characteristics of baseline and endline survey respondents

Variable	Baseline survey		Endline survey	
	Intervention	Control	Intervention	Control
	N = 188	N = 186	N = 180	N = 180
District				
Batie, n (%)	60 (32.3)	60 (31.91)	60 (33.3)	60 (33.3)
Ouargaye, n (%)	64 (34.4)	69 (36.7)	60 (33.3)	60 (33.3)
Po, n (%)	62 (33.3)	59 (31.4)	60 (33.3)	60 (33.3)
Age, in years				
Median (Range)	26 (18, 44)	27 (18, 47)	27 (18, 45)	28 (18, 48)
< 20, n (%)	26 (14.0)	28 (15.0)	25 (13.9)	21 (11.7)
20–24, n (%)	47 (25.3)	51 (27.1)	50 (27.8)	45 (25.0)
25–29, n (%)	44 (23.7)	42 (22.3)	33 (18.3)	35 (19.4)
30–34	39 (21.0)	34 (18.1)	41 (22.8)	35 (19.4)
≥ 35, n (%)	30 (16.1)	33 (17.6)	31 (17.2)	44 (24.4)
Education				
None/only religious school, n (%)	130 (69.1)	109 (58.6)	102 (56.7)	126 (70.0)
Primary/literate, n (%)	43 (22.9)	51 (27.4)	64 (35.6)	33 (18.3)
Secondary or higher, n (%)	23 (12.2)	28 (15.1)	14 (7.8)	21 (11.7)
Gravidity				
1, n (%)	42 (22.8)	53 (28.3)	41 (22.8)	39 (21.7)
2, n (%)	24 (13.0)	33 (17.6)	37 (20.6)	28 (15.6)
3, n (%)	34 (18.5)	26 (13.9)	25 (13.9)	28 (15.6)
4, n (%)	42 (22.8)	25 (13.4)	20 (11.1)	22 (12.2)
5+, n (%)	42 (22.8)	50 (26.7)	57 (31.7)	63 (35.0)
Married, n (%)	180 (96.8)	179 (95.2)	175 (97.2)	169 (93.9)
Religion				
Muslim, n (%)	77 (41.4)	73 (38.8)	73 (40.6)	62 (34.4)
n (%)	39 (21.0)	24 (12.8)	38 (21.1)	24 (13.3)
Protestant, n (%)	15 (8.1)	36 (19.2)	34 (18.9)	42 (23.3)
Traditional/animist, n (%)	45 (24.2)	51 (27.1)	28 (15.6)	48 (26.7)
None, n (%)	10 (5.4)	4 (2.1)	7 (3.9)	4 (2.2)
Work outside the home, n (%)	85 (45.7)	87 (46.3)	88 (48.9)	86 (47.8)

At baseline, women received a median of 2.1 doses (range 0–5 doses) across both arms according to documentation on the ANC card, and a median of 2.5 doses (range 0–6) according to self-report, with no significant differences across arms. At endline, control group women received a median of 1.8 doses (range 0–5) according to the card and 2.8 doses (range 0–7) per self-report and women in the intervention group received 2.8 doses (range 0–5) per ANC card and 3.4 (range 0–7) per self-report (p-value < 0.0001 for difference in arms by both card and self-report).

ANC4, IPTp3, and IPTp 4 coverage were similar at baseline, with ANC4 coverage of 61.8% in the intervention and 62.2% in the control (p-value = 0.96), IPTp3 coverage of 50.5% in the intervention and 54.3% in the control (p-value = 0.74), and IPTp4 coverage of 21.5% in the intervention and 16.0% in the control (p-value = 0.59) (Table 3).

**Table 3:** ANC and IPTp coverage at baseline and endline as documented on the ANC card, by study arm

	Baseline		Endline		Difference in differences^a^	p-value for DiD
	Control	Intervention	Control	Intervention		
	N = 188	N = 186	N = 180	N = 180		
Number of IPTp doses (mean, 95% CI)	2.3 (1.9, 2.8)	2.3 (1.8, 2.9)	2.1 (1.53, 2.9)	2.9 (2.3, 3.8)	0.9 (− 0.3, 2.1)	0.15
IPTp1+ (%, 95% CI)	86.2 (73.5, 93.4)	80.7 (65.4, 90.2)	75.6 (54.6, 88.8)	86.1 (74.9, 92.8)	16.1%-points (− 8.5, 40.7)	0.20
IPTp2+ (%, 95% CI)	73.4 (55.2, 86.1)	69.9 (57.1, 80.2)	64.4 (41.3, 82.3)	72.2 (50.3, 87.0)	11.3%-points (− 27.6, 50.1)	0.57
IPTp3+ (%, 95% CI)	54.3 (39.3, 68.5)	50.5 (35.0, 66.0)	47.2 (28.6, 66.7)	61.1 (40.6, 78.4)	17.6%-points (− 16.3, 51.5)	0.31
IPTp4+ (%, 95% CI)	16.0 (8.4, 28.3)	21.5 (8.3, 45.3)	21.1 (12, 34.5)	46.7 (29.6, 64.6)	20.0%-points (− 7.2, 47.3)	0.15
IPTp5+ (%, 95% CI)	2.7 (1.0, 6.8)	4.3 (1.1, 15.4)	2.2 (0.6, 8.1)	27.8 (17.3, 41.4)	23.9%-points (9.4, 38.4)	0.001
Number of ANC visits (mean, 95% CI)	3.2 (2.9, 3.6)	3.23 (2.9, 3.6)	3.4 (3.0, 3.7)	3.6 (3.6, 3.7)	0.2 (− 0.3, 0.8)	0.43
ANC1+ (%, 95% CI)	89.4 (80.4, 94.5)	90.3 (76.8, 96.4)	94.4 (84.7, 98.1)	97.8 (94.6, 99.1)	2.4%-points (− 10.7, 15.5)	0.72
ANC4+ (%, 95% CI)	62.2 (49.4, 73.5)	61.8 (50.5, 72.0)	65.0 (48.5, 78.6)	77.2 (73.1, 80.9)	12.6%-points (− 7.0, 3.22)	0.21

*IPTp* intermittent preventive treatment in pregnancy, *ANC* antenatal care visits

^a^Confidence interval for DiD were estimated with identity link and either binomial or poisson distribution, as appropriate

There was a 15.4 percentage point increase in the proportion of pregnant women who had attended at least four ANC visits in the intervention area, and a 2.8 percentage point increase in ANC4 attendance among women in the control group, for a DiD of 12.6 percentage points (95% CI − 7.0, 3.2). By the endline, administration of IPTp was universally higher in the intervention than control. There was an overall 17.6 percentage point increase in IPTp3 at endline in the intervention group, after accounting for baseline coverage (DiD 95% CI − 16.3, 51.5; p-value = 0.31). There was a 25.2 percentage-point increase in IPTp4 in the intervention area (p-value 0.02 for comparison from baseline to endline), and only a 5.1 percentage-point increase in the control (p-value 0.60 for comparison from baseline to endline), with a difference in differences of 20.0% (95% CI − 7.2, 47.3; p-value = 0.15). The greatest improvement was seen in IPTp5, which increased from 4.3% at baseline to 27.8% at endline in the intervention arm (DiD = 23.9 percentage points, 95% CI 9.4, 38.4; p = 0.001) (Table 3). Results did not change substantially when adjusted for maternal age and gravidity (Table 4). The calculated ICC was 0.095 for IPTp3 and 0.089 for IPTp4. Gravidity was associated with receipt of IPTp3, but not IPTp4 nor ANC attendance, while age greater than 20 was associated with ANC attendance (both ANC1 and ANC4), but not receipt of IPTp (Table 5).

**Table 4:** ANC and IPTp coverage at baseline and endline, by study arm, adjusted for gravidity and maternal age

	Baseline		Endline		Difference in differences^a^	p-value for DiD
	Control	Intervention	Control	Intervention		
	N = 188	N = 186	N = 180	N = 180		
IPTp doses (mean, 95% CI)	2.4 (2.0, 2.9)	2.3 (1.8, 3.0)	2.2 (1.6, 3.0)	3.0 (2.4, 3.8)	0.9 (− 0.3, 2.1)	0.15
IPTp3+ (%, 95% CI)	57.7 (41.6, 72.3)	53.6 (37.3, 69.1)	50.1 (30.9, 69.2)	64.2 (45.1, 79.7)	17.9%-points (− 16.4, 52.2)	0.31
IPTp4+ (%, 95% CI)	16.6 (8.6, 30.7)	22.7 (8.6, 47.8)	22.2 (12.8, 35.8)	48.5 (30.9, 66.4)	19.7%-points (− 7.4, 46.7)	0.15
ANC visits (mean, 95% CI)	3.3 (2.9, 3.6)	3.3 (2.9, 3.7)	3.4 (3.1, 3.8)	3.7 (3.6, 3.8)	0.3 (− 0.3, 0.2)	0.37
ANC1+ (%, 95% CI)	91.5 (83.2, 95.9)	92.2 (80.0, 97.2)	95.5 (87.3, 98.4)	98.3 (95.9, 98.5)	2.2%^b^	0.39
ANC4+ (%, 95% CI)	65.3 (51.4, 77.0)	64.9 (53.1, 75.1)	67.6 (50.3, 81.2)	80.2 (75.5, 84.1)	13.2%-points (− 6.0, 32.2)	0.18

*IPTp* Intermittent preventive treatment in pregnancy; *ANC* antenatal care visits

^a^Results calculated using a logistic regression model with an interaction term for survey and arm, adjusted for gravidity and maternal age (< 20 vs ≥ 20 years); Confidence interval for DiD were estimated with identity link and either binomial or poisson distribution, as appropriate

^b^The identity link model for ANC1+ did not converge, thus is was not possible to calculate a 95% CI

**Table 5:** Effects of gravidity and maternal age on uptake of IPTp and ANC

	OR	Confidence limits	p-value
IPTp doses			
Gravidity, Primi vs multi	0.84	(0.76, 0.94)	0.002
Age < 20	1.02	(0.86, 1.21)	0.78
IPTp3			
Gravidity, Primi vs multi	0.62	(0.39, 0.99)	0.05
Age < 20	0.93	(0.49, 1.77)	0.83
IPTp4			
Gravidity, Primi vs multi	0.71	(0.43, 1.17)	0.18
Age < 20	1.02	(0.65, 1.59)	0.94
ANC visits			
Gravidity, Primi vs multi	0.98	(0.9, 1.06)	0.64
Age < 20	0.92	(0.87, 0.99)	0.02
ANC1			
Gravidity, Primi vs multi	0.73	(0.36, 1.46)	0.37
Age < 20	0.58	(0.36, 0.94)	0.03
ANC4			
Gravidity, Primi vs multi	0.91	(0.51, 1.61)	0.75
Age < 20	0.61	(0.4, 0.93)	0.02

At baseline, women reported first presenting to ANC at a mean of 3.1 and 2.8 months gestational age, in control and intervention arms, respectively (p-value = 0.07); while at endline, mean gestational age at first presentation to ANC was reported to be 3.0 and 2.6 months (p-value 0.03) in control and intervention arms, controlling for age and gravidity. Among the 90.1% of women who stated that they told someone about their pregnancy, the vast majority (91.7%) disclosed that they were pregnant prior to feeling the baby moving/showing, with no significant differences between baseline/endline or control and intervention groups. Two-thirds (65.9%) reported having first told their husbands/the father of the baby, 12.1% first told their mother in law, 8.2% first told a HF health worker, and 4.9% first told their mother; only 1.8% first reported their pregnancy to a CHW.

Adjusting for age and gravidity, at baseline 34.4% and 56.7% pregnant women in the control and intervention arms, respectively, reported having spoken to a CHW about their pregnancy; this increased to 54.0% and 77.6% in the control and intervention arms, respectively, at endline. The increase was statistically significant in both arms (p-value < 0.0001 in control and p-value 0.0028 in the intervention arm; p-value 0.0002 for the difference between arms at endline). Similar proportions reported discussing prevention of MIP with a CHW (26.7% and 57.9% in the control and intervention arms, respectively, at baseline, and 51.3% and 74.3% in the control and intervention arms, respectively, at endline). The increase was statistically significantly different in the control (p-value 0.007) but not the intervention arm (p-value 0.13), however, given the difference at baseline there was still a statistically significant difference between arms at endline (p-value 0.01).

## Discussion

Despite a WHO recommendation for all pregnant women to receive at least three doses of IPTp, coverage in most of sub-Saharan Africa remains below international targets [6]. This study demonstrates that CHWs can effectively deliver IPTp, without adversely affecting ANC attendance. Further, not only did community delivery of IPTp not lead to a reduction in ANC attendance, but retention in ANC improved over the course of the study, with 12.6 percentage point increase in ANC4 in the intervention arm compared to the control arm (95% CI − 7.0, 32.2). More than half of the IPTp doses were delivered by the CHWs, highlighting their substantial contribution to the increased coverage. The increase in coverage of ANC4 further highlights the important community education and outreach role of these workers.

Concerns have been raised that providing IPTp in the community could lead to women receiving many more IPTp doses than are currently recommended. By requiring women receive the first dose of IPTp-SP at the HF, this study ensured that women were not started on IPTp until they were deemed eligible, at least 13 weeks. Prior to implementation of the study, a small proportion of women reported that they had received 6 doses of IPTp. At endline, there was a small and insignificant increase in women receiving 6 doses, and only a very few women (n = 6) who reported having received 7 doses, highlighting that IPTp was being correctly given according to the monthly schedule. Further, it was apparent from review of the CHW monthly data that most women had delivered by the time they were due for IPTp5. As the card only had space to record five doses of IPTp, it is possible that the discrepancy in self-reported and documented IPTp truly reflects that women were receiving six and seven doses—a woman who received her first dose at 13 weeks and then continued to receive doses every 4 weeks thereafter could reach as many as eight doses.

Despite the substantial increases in IPTp coverage, there was not a statistically significant DiD for any of the measures. This was likely due to insufficient power, as the study was powered on prior estimates of IPTp coverage that were lower than what existed at the time the study began, and assumed a lower ICC than existed. However, the fact that there was an improvement in retention in ANC should alleviate many of the concerns around community delivery of IPTp. While further, larger studies are warranted to confirm that this method of delivery results in significant improvements, this study suggests that there is no disadvantage to community IPTp distribution, with no adverse events reported as a result of SP administration by CHWs.

The importance of early attendance at ANC cannot be overstated. Late attendance at ANC has been associated with an increased risk of maternal and fetal complications, including low birth weight, premature delivery, and stillbirth [17, 18]. Early initiation of ANC is crucial to ensure pregnant women receive health education and preventive services, such as iron supplementation, tetanus injection, and deworming, as well as early implementation of IPTp, which has been shown to have a greater impact than later administration [19–22]. Engaging CHWs in identifying pregnant women in the community and encouraging them to attend ANC early has the potential to improve coverage of early ANC. At endline, women in the intervention arm started ANC significantly earlier than women in the control arm, suggesting an impact of the intervention. While a large proportion of women spoke to a CHW about their pregnancy, there is still room for improvement, as even in the intervention area, 24% of women reportedly spoke with neither; engaging these women could have substantially boosted coverage.

Our results suggest that women recognize very early in pregnancy that they are pregnant, and that they disclose their pregnancies most often to their husbands or other family members. This suggests that messaging around the importance of attending ANC early needs to target not only the pregnant woman, but also their husbands and families, to encourage them to attend early, as has been suggested previously [23–25]. In Ethiopia, male involvement in ANC was positively associated with both ANC attendance in first trimester and delivery in a HF [24].

c-IPTp may be a promising new channel for scaling up and closing the remaining coverage gap of IPTp, particularly in remote rural areas and in high malaria burden countries. WHO recognizes that while some countries have experienced a reduced burden of malaria, progress in high-burden countries such as Burkina Faso has stalled in recent years. In 2016, there were an estimated 216 million cases of malaria globally, marking a return to 2012 case levels, with an increase to 219 million cases in 2017 [6, 26]. WHO points to persistent coverage gaps of proven prevention and control interventions in high burden countries, due in part to weak health systems. According to WHO’s High-Burden to High Impact Initiative: national data from many high-burden countries show coverage gaps for the core malaria interventions (i.e. vector control, case management, and IPTp) [27]. In 2016, approximately 80% of eligible pregnant women across sub-Saharan Africa did not receive the recommended three or more doses of IPTp [27]. Implementation of c-IPTp could reduce the number of pregnant women in sub-Saharan Africa who are not offered the third dose of IPTp during their pregnancy.

While c-IPTp is not the only solution to improving IPTp uptake, it has several advantages, including shifting the task of providing IPTp from already overburdened ANC facility staff to trained CHWs, allowing ANC nurses to spend more time with their clients providing a positive pregnancy experience—a key recommendation of the 2016 WHO guideline on ANC, providing “pregnant women with respectful, individualized, person-centred care at every contact” [28]. While task-shifting components of ANC health promotion to a broad range of cadres, the WHO recommendation for delivery of IPTp falls short of recommending task-shifting to lay health workers, including only “auxiliary nurses, nurses, midwives and doctors.” This study’s findings suggest that WHO’s recommendations on task shifting of IPTp could be expanded to include lay health workers; offering an alternative approach that increases patient access to services and enhances efficiency of the ANC services package. Ample data exist from malaria diagnosis and treatment studies highlighting CHW feasibility and capabilities in effectively providing malaria treatment, accurately diagnosing fever following RDT results, and providing seasonal malaria chemoprophylaxis to children under 5 years of age [29–31]. Several studies, including this one, demonstrated that CHWs can contribute to improved ANC attendance and ANC attendance is not negatively affected by community delivery of IPTp, with no reported increases in adverse events [7, 8, 11].

## Conclusions

To achieve the ambitious targets for malaria eradication by 2050 [32], rapid accelerated coverage of proven interventions is needed. Every available tool in our toolbox must be used, including engaging communities in this effort and allowing CHWs to deliver malaria prevention and control interventions. After decades of promoting IPTp, coverage of this intervention remains among the lowest, with an estimated 22% of pregnant women receiving the recommended three doses [6]. Implementation of community-IPTp provided by trained CHWs could improve coverage and access for millions of pregnant women across sub-Saharan Africa. With an estimated 215,000 LBW deliveries annually as well as up to 20% of stillbirths in sub-Saharan Africa which could be prevented by complete scale-up of IPTp, the time to act is now [33, 34].

## Data Availability

Data has been made available on the USAID Development Data Library (DDL; https://data.usaid.gov/) as restricted access pending approval from Burkina Faso government. Data can also be requested from Jhpiego by writing to opendatahelp@jhpiego.org under a data use agreement.

## Abbreviations

ANC: Antenatal care
CHW: Community health worker
CI: Confidence interval
c-IPTp: Community level distribution of intermittent preventive treatment of pregnant women
DiD: Difference in differences
HF: Health facility
IPTp: Intermittent preventive treatment of pregnant women
LLIN: Long-lasting insecticide-treated nets
MIP: Malaria in pregnancy
MOH: Ministry of Health
SP: Sulfadoxine pyrimethamine
WHO: World Health Organization
